# A distinctive “Shotgun-like” ^18^F-FDG PET uptake pattern in disseminated cutaneous sarcoidosis responding to TNFα inhibitors

**DOI:** 10.1007/s00259-026-07791-9

**Published:** 2026-02-03

**Authors:** Roberto Colli, Jakob Nilsson

**Affiliations:** Department of Immunology, University Hospital Zurich, Schmelzbergstrasse 26, 8091 Zurich (CH), Switzerland

A 36-year-old woman, formerly chimney sweeper, was referred to our hospital with a 10-year history of progressive, painful-itchy cutaneous lesions over the entire body surface and night sweats. Clinical examination revealed different stages of cutaneous lesions on the entire skin surface and the scalp, ranging from raised nodules to circular ulcerations and open wounds up to 4–5 cm in size (Figure A). Elevated serological markers of granulomatous inflammation Neopterin, sIL-2R and TNFα, along with a slightly elevated CRP and normal blood cell counts were noted. PET scan revealed a distinctive ^18^F-FDG-uptake pattern with “shotgun-like” disseminated lesions across the entire cutaneous surface (Figure B). Nodular and ulcerating lesions showed high tracer uptake, with SUVmax values up to 24.5 on the scalp and 12 on the calf (Figure C). Additional findings included ^18^F-FDG-avid involvement of bone and spleen and lymphadenopathy in multiple nodal stations.

Consistent with the elevated blood inflammatory markers, histology of different lesions revealed typical non-necrotizing granulomas. After exclusion of infectious causes, a diagnosis of cutaneous sarcoidosis was established. Treatment with the anti-TNFα antibody adalimumab (80 mg weekly) and prednisone (10 mg daily, tapered over 12 weeks) was then initiated.

After 6 months, a robust clinical response was observed, with healing of cutaneous lesions (Figure D) and marked improvement of the ^18^F-FDG-PET lesions (Figure E).

This case highlights an unusual, yet clinically recognizable presentation of diffuse cutaneous sarcoidosis. Prior occupational exposure to combustion products may have contributed to the diffuse spread of disease. On PET, this translated into a characteristic whole-body “shotgun-like” 18F-FDG uptake pattern, highly responsive to TNFα-inhibitor therapy. Recognition of this PET appearance in sarcoidosis is relevant for differential diagnosis in patients presenting with diffuse nodular ^18^F-FDG avid lesions, as it may mimic metastatic, lymphoproliferative or disseminated infectious diseases.

Previous reports have described ^18^F-FDG PET findings in micronodular [1] and scaling [2] forms of cutaneous sarcoidosis. To our knowledge, this is the first ^18^F-FDG PET-characterized case with diffuse ulcerating macro-nodular lesions. The correlation between the impressive clinical pictures and the macronodular “shotgun-like” appearance on PET, together with the marked response to biologic therapy with TNFα-inhibitors, may contribute novel, clinically relevant insights into the differential diagnosis and management of uncommon cutaneous manifestations.

**Figure:**
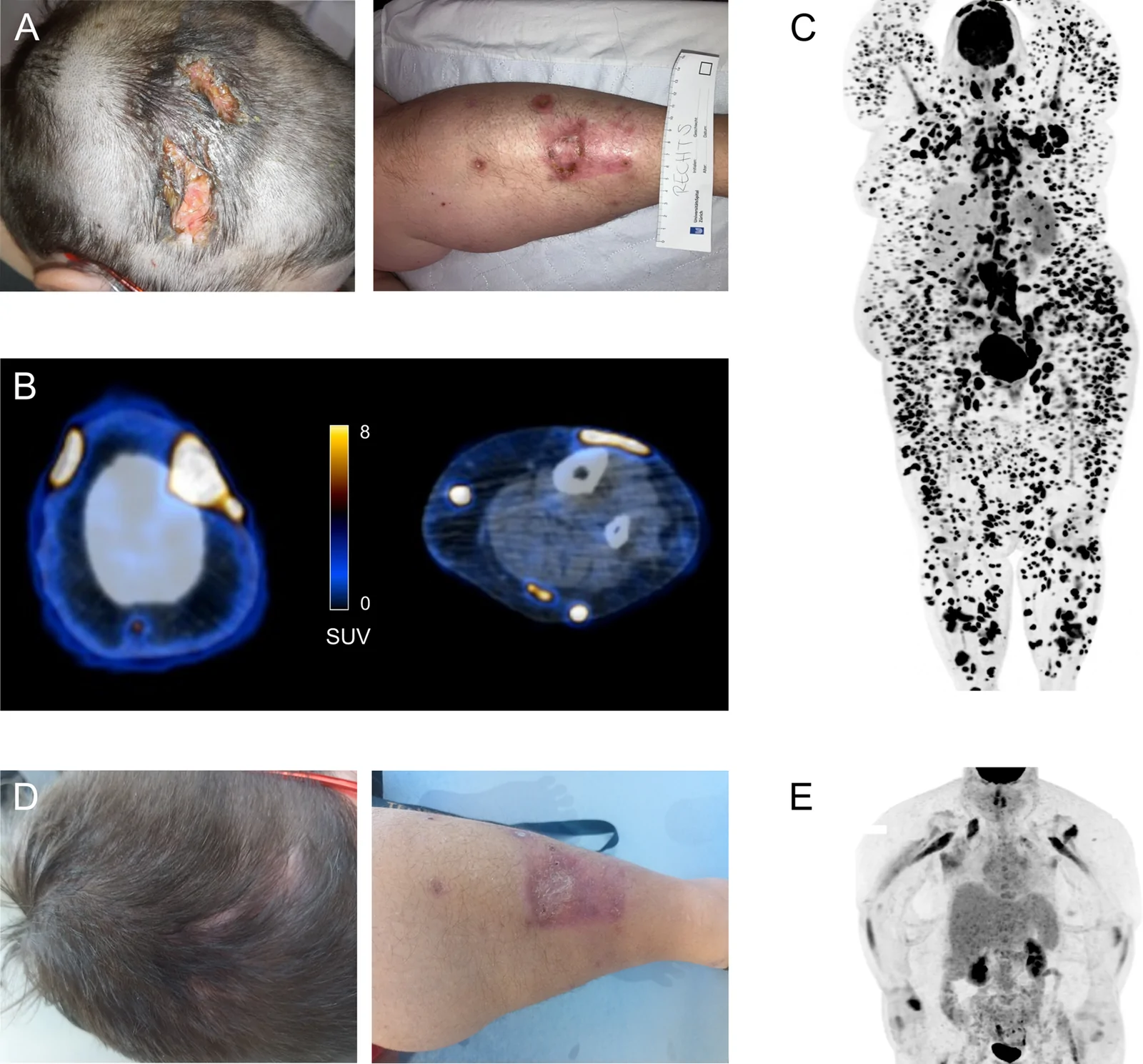

## Data Availability

Data sharing not applicable to this article as no datasets were generated or analysed during the current study.
